# Incidental lymphadenopathy in renal transplantation

**DOI:** 10.1308/rcsann.2023.0043

**Published:** 2023-07-25

**Authors:** NX Ho, AK Malik, S Moulding, F Farrow, D Talbot, S White, D Rix, G Sen, D Manas, A Amer, R Figuereido, CH Wilson

**Affiliations:** The Newcastle upon Tyne Hospitals NHS Foundation Trust, UK

**Keywords:** Lymphadenopathy, Renal transplantation, Iliac lymphadenopathy, Kidney transplant, Lymph node biopsy

## Abstract

**Introduction:**

Iliac lymphadenectomy is performed to provide anastomotic access during the vascular implantation procedure in renal transplantation. Iliac lymph nodes (LNs) are often enlarged, but there are no standardised guidelines for the management of incidentally enlarged LNs during transplantation. We aimed to evaluate histological findings of LNs sent for examination at our unit.

**Methods:**

Patients were evaluated in two distinct date cycles. In the first cycle, lymphadenectomy and histological assessment were performed at the discretion of the transplanting surgeon. In the second cycle, all incidentally enlarged LNs were sent for histological assessment, regardless of size.

**Results:**

In the first cycle (*n* = 76), 11 patients (14.47%) had incidentally enlarged iliac LNs on lymphadenectomy and histology showed only reactive changes. In the second cycle (*n* = 165), eight patients (4.85%) had incidentally enlarged LNs on lymphadenectomy. One patient was found to have mature B cell chronic lymphocytic leukaemia. The patient was referred to haematology and a “watch and wait” approach was taken, with the patient still alive at last follow-up (511 days post-transplantation).

**Discussion:**

There are currently no published guidelines on the management of incidentally enlarged iliac LNs during transplantation. Current literature suggests that clinically significant lymphadenopathy needs to be investigated in all patients. Based on our centre’s experience of a 5.26% (1 in 19) positive pathological LN sampling, we recommend that all incidental LNs with suspicious features and/or that are greater than 10mm in diameter should be considered for histological, microbiological and molecular assessment as appropriate.

## Introduction

Renal transplant remains the mainstay of treatment for patients with end-stage renal disease (ESRD).^1^ Results have improved dramatically over the past 50 years to the point that graft and patient survival are now over 90% in most countries around the world.

Iliac lymphadenectomy is performed to provide adequate access for anastomosis during the implantation procedure.^2^ Often these lymph nodes (LN) are enlarged, but there are no standardised guidelines on the management of incidentally enlarged iliac LN at the time of transplantation. We aimed to evaluate the histological findings of LNs sent for examination in our institution and the significance of performing immunohistochemical analysis of incidentally enlarged LNs.

## Methods

Histopathology records of consecutive patients for transplantation were reviewed. LNs that were biopsied underwent initial macroscopic inspection and subsequent haematoxylin and eosin staining for microscopic review. Further immunohistochemistry and in situ hybridisation were done to characterise any positive findings from microscopy.

Patients were evaluated in two distinct date cycles. The first cycle was from 1 January to 31 August 2020, and the second cycle was from 1 September 2020 to 1 October 2021.

In the first cycle, selective lymphadenectomy and histological assessment were performed at the discretion of the transplanting surgeon. In the second cycle, all incidentally enlarged LNs were sent for histological assessment, regardless of size.

## Results

In the first audit cycle, a total of 76 patients underwent renal transplantation of whom 11 (14.47%) had incidentally enlarged iliac LNs at the time of transplantation, with an excision biopsy performed (Table 1). Excision biopsies showed benign reactive changes (Table 2). After the first cycle, our unit implemented a guideline that all patients with incidentally enlarged iliac LNs at renal transplantation should undergo biopsy and assessment.

**Table 1:** Summary of audit findings

Audit cycle	First audit cycle (January to August 2020)	Second audit cycle (September 2020 to October 2021)
Total no. of patients	76	165
Gender	Male 27	Male 84
	Female 49	Female 81
Median age; years	55.5	53.5
No. of patients with incidentally enlarged iliac lymph nodes	11	8
Median diameter of incidentally enlarged lymph nodes; mm	30 (12–77)	40 (13–59)

**Table 2:** Histopathological outcome of lymph node biopsy

Outcome of lymph node excision biopsies	First audit cycle (January to August 2020)	Second audit cycle (September 2020 to October 2021)
	All 11 biopsies showed reactive changes only	5 biopsies showed reactive changes
		In 2 biopsies the sample did not contain any lymph nodes
		1 biopsy was positive for mature B cell chronic lymphocytic leukaemia (lymph nodes sized at 35mm and 45mm)

**Figure 1:**
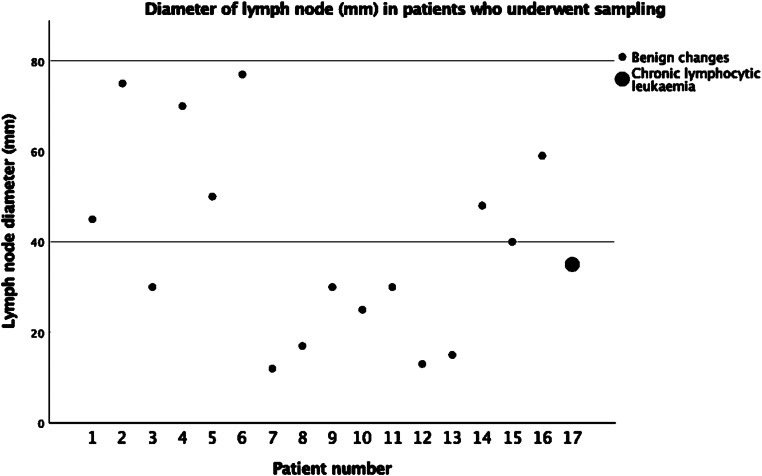
Scatter plot of lymph node diameters in patients who underwent biopsy

During the second cycle, 165 patients underwent renal transplantation; 8 (4.85%) had incidentally enlarged iliac LNs on lymphadenectomy (Table 1). Excision biopsies were carried out on all eight patients, as detailed in Table 2. There was no history of preoperative sepsis in any of the patients with incidentally enlarged LNs. One patient with a 35mm LN was found to have mature B cell chronic lymphocytic leukaemia (CLL) on biopsy (Figure 1).

Further evaluation was carried out on this patient with a follow-up bone marrow biopsy, which confirmed the diagnosis of a new CLL. Staging computed tomography further demonstrated lymphadenopathy on both sides of the diaphragm, as well as an incidental renal cell carcinoma on their native right kidney. The lesion was suboptimal for biopsy, lying near the hilum of the right kidney, therefore diagnosis was based on radiological findings alone.

The patient was referred to the haematology team and a “watch and wait” approach was opted for in this case, with the patient still alive at last follow-up (511 days post-transplantation). Standard maintenance immunosuppression is with mycophenolate mofetil (MMF, starting at 2g a day reducing to 1g), tacrolimus (dose adjusted according to plasma levels) and 5mg prednisolone. On discussion with the haematology team, MMF was reduced to 250mg once daily. Following imaging suggestive of renal cell carcinoma, MMF was completely stopped.

## Discussion

There are currently no published guidelines on how patients with incidental iliac LNs at the time of renal transplantation should be managed. A case report by Terada *et al* described a rare incidence of unexpected CLL at the time of lung transplantation, in which incidentally enlarged right hilar LNs were identified and biopsied during transplantation.^3^ The patient subsequently underwent concurrent tyrosine kinase inhibitor therapy and maintenance post-transplantation immunosuppression with a good clinical outcome and stable organ function thereafter. In our series, the incidental finding of CLL resulted in a reduction in the immunosuppression regimen to prevent rapid proliferation of the disease.

There is no doubt that clinically significant lymphadenopathy needs to be investigated in all patients – independent of transplant status. In a 1966 study by Ellman *et al*, all enlarged, superficial LN biopsies were reviewed over a two-year period (*n* = 135) and 63% showed a significant finding at pathology. Of these biopsies, 50 were positive for lymphoma, 14 had carcinoma, 7 suppurative lymphadenitis, 6 tuberculosis, 1 had histoplasmosis and 1 showed diphenyl-hydantoin induced lymphadenopathy.^4^

A more recent study has demonstrated that lymphoma is a common cause of lymphadenopathy and that all suspicious LNs should be biopsied, given the limitations of fine-needle aspiration cytology techniques.^5^ The retrospective study reviewed all cases of LN biopsies over a five-year period and lymphoma was confirmed in 62 (20.88%) of the 297 patients in the study population. In addition, the most recent European Society of Medical Oncology guidelines on the diagnosis of CLL recommends that LN biopsy and/or bone marrow biopsy may be helpful when immunophenotyping.^6^

The most widely accepted method to distinguish between pathological and benign LNs is by visual assessment of size. Current literature reports have noted reasonable variation in the upper limit of LN sizes across different anatomical sites. For instance, the upper limit for normal pelvic nodes has been described as being between 8 and 10mm, and anything greater than 10mm would be considered pathological.^7^ Guidelines in the assessment of LNs in non-Hodgkin’s lymphoma consider any LN greater than 15 mm in greatest transverse diameter as abnormal.^8,9^

Pathologically enlarged LNs could also indicate the presence of infection. Active infections in the pretransplant recipient should be eliminated before transplantation, as immunosuppression post-transplantation will exacerbate and/or reactivate underlying infections. As such, recipients are routinely evaluated for active infection and risk factors for infection through serological and radiological screening tests, in addition to thorough clinical history and physical examination.^10–12^ Furthermore, patients awaiting renal transplantation commonly have either a haemodialysis and/or peritoneal dialysis access site, which acts as a risk for source of infection.

A study by Woeltje *et al* described that patients with ESRD are 16 times more likely to develop active *Mycobacterium* tuberculosis (TB).^13^ In addition, a high proportion of ESRD patients display anergy and this would warrant further clinical evaluation of TB in the context of negative tuberculin skin tests.^14^ A recent review by Ganchua *et al* described LNs as one of the most common sites of extrapulmonary TB, in which there is disruption in the normal cellular architecture of LNs, where evidence of granuloma formation can be seen in histopathological analysis.^15^ Diagnosis of tuberculosis through *Mycobacterium* culture remains challenging owing to its indolent growth in Lowenstein–Jensen medium, requiring several weeks of culture before results are available, leading to delays in diagnosis.^16,17^ Studies have shown that polymerase chain reaction analysis is a useful tool in diagnosing TB and is reported to be effective and superior in terms of speed of diagnosis.^18^

## Conclusions

In general, a systematic approach to lymphadenopathy should be adopted in the clinical setting, and correlation with radiological findings and relevant past medical and/or social history should be taken into account when enlarged LNs are encountered. Microbiological assessment of the biopsy sample should be considered if there is any suspicion that the lymphadenopathy is a consequence of an infection. The presence of granulomatous disruption within the cellular architecture of biopsied LNs indicates extrapulmonary TB infection, and warrants further microbiological and molecular analysis for diagnosis of the disease.^19^

There is no current consensus on the standard approach for incidentally enlarged LNs during organ transplantation. In our series, a positive finding altered management with a reduction in immunosuppression, which may not have occurred until much later following disease proliferation without biopsy. Based on our centre’s experience of a 5.26% (1 in 19) incidence of positive pathological findings following LN sampling, we recommend that all incidental LNs with suspicious features and/or that are greater than 10mm in diameter should be considered for histological assessment. Although microbiological assessment was not included in our series, we recommend further evaluation of suspicious LNs with microbiological and molecular testing, should there be any clinical indication of latent infections such as tuberculosis, in line with recommendations from current literature.
